# Primary Cilia and Cardiovascular Risk Factors in Alzheimer’s Disease

**DOI:** 10.3390/brainsci15091004

**Published:** 2025-09-17

**Authors:** Clare L. Sunderman, Kathleen V. Forero, Qasim Alorjani, Raghad Buqaileh, Gillian M. Gallagher, Sestina M. Ventresca, William S. Messer, Wissam A. AbouAlaiwi

**Affiliations:** Department of Pharmacology and Experimental Therapeutics, University of Toledo, Toledo, OH 43614, USA; csunder@rockets.utoledo.edu (C.L.S.); kathleen.forero@rockets.utoledo.edu (K.V.F.); qasim.alorjani@rockets.utoledo.edu (Q.A.); raghad.buqaileh@rockets.utoledo.edu (R.B.); gillian.gallagher@rockets.utoledo.edu (G.M.G.); sestina.ventresca@rockets.utoledo.edu (S.M.V.); william.messer@utoledo.edu (W.S.M.J.)

**Keywords:** Alzheimer’s disease, primary cilia, nitric oxide, cardiovascular disease

## Abstract

Alzheimer’s disease (AD) is the most common cause of dementia worldwide due to an aging population. AD is characterized as a progressive neurodegenerative disease that leads to atrophy of brain tissue, causing cognitive deficits. Amyloid beta plaques and neurofibrillary tangles are pathological hallmarks of AD, yet the cause is still highly debated. Many other cardiovascular diseases and vascular manifestations share the same symptoms as patients with AD. In this review, the current understanding of AD is summarized with a brief discussion on how primary cilia dysfunction and impaired nitric oxide (NO) signaling contribute to cardiovascular risk factors, vascular pathology, and cognitive decline in AD. Finally, we highlight primary cilia as a possible therapeutic target and any future directions for treating AD.

## 1. Introduction

As of this report, approximately 6.7 million Americans have been diagnosed with Alzheimer’s disease (AD), with a prevalence of 1 in 9 individuals who are 65 years and older [1]. AD is the most common form of dementia, which is a general term for cognitive deficit symptoms. It is hypothesized that the AD process begins about 20 years before the clinical onset of dementia [2]. AD is characterized as a progressive neurodegenerative disease that leads to atrophy of brain tissue. The two hallmark biomarkers of AD are amyloid beta (Aβ) plaque accumulation and neurofibrillary tangles. Amyloids are a type of protein prone to structural conversion and assemble into cross-β-structured fibrils [3]. Aβ peptides are formed by proteolytic cleavage of the amyloid precursor protein (APP), specifically the hydrophobic transmembrane portion, by β- and γ-secretases [3]. Aβ plaques are accumulations of sticky Aβ peptides outside of the neurons and impede the synaptic communication between neurons, leading to neuronal death. Neurofibrillary tangles are accumulations of excessive hyperphosphorylated tau proteins within neurons and impact the movement of nutrients and halt normal neuronal function. The progressive neuronal death leads to a decrease in brain mass that can be detected through imaging techniques such as MRI. This loss of brain mass can lead to various cognitive deficits, including difficulty with memory, planning, and judgment, and they appear initially as mild deficits, yet compound with age.

While Aβ and tau remain defining hallmarks of AD, recent reports suggest they may represent only part of a broader and more complex pathophysiology. Increasingly, AD is being reframed as a systemic disorder linked to global aging processes rather than a purely localized brain disease [4,5]. Systemic aging is considered a major driver of vulnerability, and AD shares mechanistic overlap with other neurodegenerative disorders such as Parkinson’s disease and amyotrophic lateral sclerosis, particularly through two complementary mechanisms: the synaptic spread hypothesis, where pathogenic proteins propagate from neuron to neuron across synapses and the selective vulnerability hypothesis, where certain neuronal populations degenerate earlier due to intrinsic susceptibilities like metabolic stress. While awaiting definitive evidence, many researchers currently consider both mechanisms as coexisting factors in disease progression [6]. A new perspective on AD suggests that metabolic dysfunction, mitochondrial dysfunction, and oxidative stress have been identified as early contributors to AD, with amyloid and tau accumulation seen as later outcomes of wider metabolic and vascular disturbances [7]. A new perspective on AD mechanisms highlights the role of the lipid invasion model in AD, which proposes that breakdown of the blood–brain barrier permits harmful lipids like low-density lipoprotein and free fatty acids with inflammatory mediators to enter the brain, thereby triggering and fueling amyloidogenesis and tau pathology [8]. Recently, neuroinflammation, especially through microglial overactivation and the nucleotide-binding domain, leucine-rich–containing family, pyrin domain–containing-3 (NLRP3) inflammasome, has gained recognition as a major driver of neuronal damage in AD, fueling a destructive cycle of reduced amyloid clearance and ongoing degeneration [9]. The immune system may also play a role in the pathological process of AD. Experimental evidence has demonstrated that immune cells may undergo significant changes and perform different functions at various stages of the disease [10]. These emerging insights align with the wider neurodegeneration field and underscore the need for a more cohesive mechanistic framework for AD.

APP moderates cell growth, motility, and survival, as well as neurite outgrowth and functions, but in the disease state, APP produces amyloidogenic fragments through differential cleavage by Beta-Site Amyloid Precursor Protein Cleaving Enzyme 1 (BACE-1) [11,12]. The two main Aβ polymers involved in plaque formation are Aβ_40_ and Aβ_42_. Aβ_42_ is more susceptible to aggregation, but aggregation of both can result in blocked ion channels, altered calcium (Ca^2+^) homeostasis, and decreased energy metabolism and glucose regulation, all of which aid in the deterioration of neuronal health [13].

Although AD is characterized as a neurodegenerative disease, there are certain areas of the brain that are impacted more than others. The hippocampus, a portion of the temporal lobe that is important in the formation of new memories, is an area of the brain that degenerates early in AD pathology [14]. Another area impacted in the early stages is the entorhinal cortex, which is involved in the formation of long-term memory. In the later stages, the disease impacts the medial and inferior temporal lobe, such as the middle temporal gyrus, which regulates semantic memory and language processing, and then later the primary sensory and motor cortex [15]. Understanding the pathophysiology of brain degeneration in AD helps to understand the progression of symptoms as the disease state worsens.

**Figure 1:**
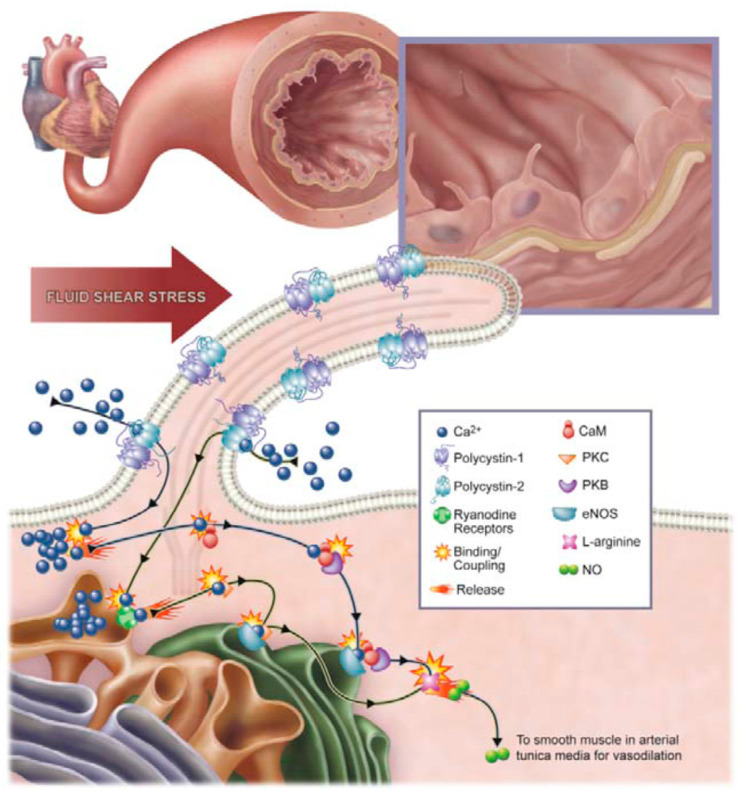
Primary vascular endothelial cilia play a role in the biochemical production of nitric oxide (NO), a potent vasodilator. The figure illustrates how NO is produced in an artery. In physiological conditions, a rise in blood pressure will be detected through the mechanosensory role of primary cilia as a result of increased vascular shear stress. The bending or activation of these cilia involves the mechanosensory polycystin-1 and polycystin-2 complex, triggering a signaling cascade that leads to NO production. This cascade includes an influx of extracellular calcium ions (Ca^2+^), which subsequently activates several calcium-dependent proteins, such as calmodulin (CaM) and protein kinase C (PKC). Along with protein kinase B (PKB), CaM and PKC act as key downstream effectors that activate endothelial nitric oxide synthase (eNOS), the enzyme responsible for NO synthesis. Figure is adopted with permission from [21].

Studying the comorbidities associated with AD is also important to better understand the pathology of AD and improve future treatments. Associations have been documented between AD and different disease states, such as gastrointestinal disorders, diabetes, depression, and cardiovascular diseases [16,17,18,19]. In this context, primary cilia have emerged as important signaling hubs, and dysregulation of pathways coordinated through them, such as Sonic hedgehog (Shh), may contribute to impaired neuronal function in AD [20]. Alongside nitric oxide (NO) signaling, which plays a dual role in vascular regulation and oxidative stress, ciliary dysfunction may represent a mechanistic link between AD and its cardiovascular comorbidities. In this review, we highlight primary cilia and NO (Figure 1) and how they contribute to comorbidities of AD, specifically with respect to cardiovascular diseases. Although the mechanism of pathology between cilia and AD has not been identified, studying the associations can lead to improved therapeutic approaches.

## 2. Primary Cilia and Ciliary Receptors

Cilia are hair-like structures that project from the surface of various cell types in the body, including stem, neuronal, endothelial, and epithelial [22]. A more detailed list of specialized cell types and their ciliary functions can be found in Table 1.

**Figure 2:**
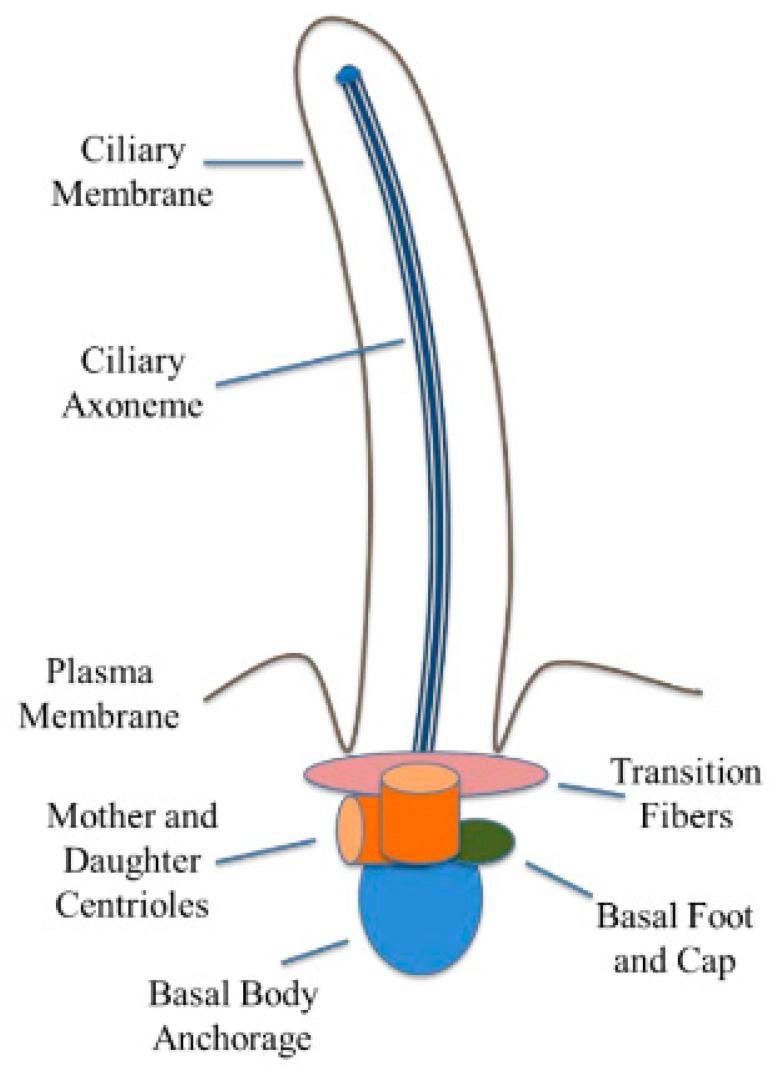
Illustration of the structure of primary cilia. The primary cilia are made up of the ciliary membrane, axoneme, and basal body. The ciliary membrane contains protein receptors and channels responsible for proper cilia function. The axoneme forms the ciliary skeleton, while the basal body is anchored to the plasma membrane with the help of the centrioles. Figure is adopted with permission from [50].

They are classified into two categories: motile and non-motile. Motile cilia contain dynein motor structures that allow them to move, while non-motile cilia lack these structures [43]. Non-motile cilia, also known as primary cilia, act as sensory organelles that help regulate signaling pathways. Primary cilia lack a central pair of microtubules and exist as monocilia on the surface of cells (Figure 2) [43].

The ciliary membrane has a variety of localized receptors and channels that make its predominant function the detection, transmission, and translation of external mechanical or chemical stimuli [44]. One signaling pathway that primary cilia regulate is the Hedgehog pathway (Hh). The Hh is used frequently for intercellular communication in development, but is entirely dependent on primary cilia [45]. The three mammalian Hh proteins are Sonic (Shh), Indian, and Desert, with Shh being the focus with its role as a morphogen and inducing various cell types at different concentration thresholds [46]. In relation to AD, it has been shown that Aβ deposition can cause cilia length to shorten in vitro and in vivo [47,48]. It has been postulated that increased Aβ leads to cilia degeneration, which inhibits Shh [49]. The shortening of cilia can impair their ability to sense and regulate external signals, leading to the promotion of AD [47].

Primary endothelial cilia are normally found in areas of low blood flow, suggesting that the cilia play a role in signal amplification of fluid shear forces into the cell [51]. Primary cilia have an active role in fluid shear stress. Fluid shear stress occurs when viscous blood imposes a drag force on endothelial cells, which acts on the vascular walls in parallel to the blood flow [51]. When shear stress is activated, the endothelial cells regulate the expression of genes responsive to shear. This regulation activates vasoactive genes like endothelial nitric oxide synthase (eNOS) and adjusts the activation state of cells to normalize shear stress [51]. These cilia react to changes in blood pressure and activate pathways that regulate the synthesis and release of NO [52]. When impaired cilia fail to sense fluid shear stress, endothelial biosynthesis of NO is reduced, which can potentially lead to increased blood pressure (Figure 1).

Primary cilia are also found on most neurons in the brain. These neuronal cilia sense neuromodulators in the surrounding environment and influence neuronal functions by contacts it makes with synapses, axonal segments, and other cells [53]. By extending into the synaptic cleft, neuronal cilia expose their membrane receptors, which mediate the release and sensing of neurotransmitters and neuromodulators [53]. These neurotransmitters, such as serotonin, dopamine, and norepinephrine, can activate various G-protein-coupled receptors (GPCRs) that are expressed in the cerebral cortex. Recent studies show that neuronal primary cilia form specialized axo-ciliary synapses and host GPCRs (e.g., somatostatin receptor type 3 (SSTR3) and 5-hydroxytryptamine receptor 6 (5-HT6)) that tune synaptic plasticity and circuit excitability. In AD models, ciliary signaling and dynamics are perturbed and linked to defects in dendritic integration, connectivity, and memory function [54]. In parallel, recent reports also implicate endothelial primary cilia as shear-stress mechanosensors that regulate NO bioavailability, barrier integrity, and neurovascular coupling; work in AD models and patient-relevant systems associates endothelial cilia dysfunction with early neurovascular decoupling and cerebrovascular instability [52,55,56]. Another type of primary cilia is astrocytic primary cilia, but not as much is known about them. There is evidence that astrocytic primary cilia play a role in brain tumorigenesis [29]. Glioblastomas are brain tumors that arise from the irregular proliferation of astrocytes and are highly related to malformed primary cilia [29]. In samples of glioblastomas, defects in the beginning stages of ciliogenesis of astrocytes were identified.

There are a variety of ligand-receptor complexes on the ciliary membrane, one being the GPCRs. GPCRs are the largest family of membrane proteins in the human genome, with over 800 unique members, and are the largest family of targets for approved drugs [57]. GPCRs convert extracellular signals, including neurotransmitters, peptides, ions, odorants, and light, into intracellular effects through conformational changes that allow for the recruitment and activation of a G-protein, inducing a signaling cascade [58]. Dysregulation of GPCR signaling in cilia has been associated with numerous neurological disorders [59].

Muscarinic acetylcholine receptors (MR) are among the GPCRs most associated with AD. Their involvement in cognitive decline was first linked to disrupted cholinergic signaling caused by the loss of cholinergic neurons [60]. This, coupled with the association of cholinergic signaling with higher-order brain functions, including learning, cognition, and memory, gave way to the cholinergic hypothesis of AD [61]. Restoring cholinergic signaling through acetylcholinesterase inhibitors (AChEIs), which inhibit the breakdown of acetylcholine (ACh), remains a first-line treatment strategy for AD. AChEIs, including the FDA-approved donepezil, galantamine, and rivastigmine, can relieve cognitive dysfunction and reduce cognitive decline in mild to moderate AD patients [60,61]. However, the utility of AChEIs is limited by adverse effects stemming from the nonspecific up-regulation of cholinergic signaling across the whole body [61]. Furthermore, the effects of AChEIs on cognition are generally only modest, and AChEIs do not treat the underlying causes of AD or modify the disease in any clinically meaningful way [62].

Muscarinic acetylcholine receptors have been the target of numerous preclinical and clinical efforts for treating AD. There are five known subtypes of muscarinic receptors all of which are distributed throughout the central nervous system (CNS) and periphery and mediate a wide range of physiological functions. The muscarinic receptor 1 (M1R) subtype has been of particular interest for its relation to AD, as the M1R accounts for 50–60% of all muscarinic expression in the CNS. It is particularly abundant in all major areas of the forebrain, including the hippocampus, neostriatum, cerebral cortex, corpus striatum, and thalamus [61,63]. Of all the muscarinic receptors, the muscarinic receptor 3 (M3R) has the lowest expression in the CNS but is highly expressed in the hypothalamus relative to the other subtypes [61,63]. Compared to many of the other muscarinic receptors, the functions of the M3R in the CNS are unclear [61]; however, phosphorylation of the M3R has been associated with learning and memory [64]. Although not in high quantities in the CNS, M3R was demonstrated to be localized to primary endothelial cilia [52]. Along with localization, the M3R is involved in altering cilia length and sensory function. Treatment with a M3R agonist significantly increased cilia length in wild-type endothelial cells, while treatment with an M3R antagonist significantly decreased cilia length and the percentage of ciliated cells [52]. It was proposed that M3R-mediated changes in cilia length in endothelial cells could alter the function of primary cilia, leading to behavioral changes in organisms, though the exact mechanism has yet to be identified. Concomitant with M3R-mediated increase in cilia length, there is also an upregulation in phosphorylated endothelial nitric oxide synthase (p-eNOS) in endothelial cells [52]. Thus, it has been hypothesized that impaired ciliary M3R signaling could lead to decreased NO availability [52].

## 3. Nitric Oxide and Alzheimer’s Disease

Oxidative stress is associated with an imbalance in the production and accumulation of reactive oxygen species (ROS) in cells, as well as the capacity of the body to detoxify the products with antioxidants [65]. ROS are mainly produced in mitochondria during various processes like protein phosphorylation, apoptosis, and immunity, which are all dependent on ROS production [65]. But an overload of ROS can lead to the onset or progression of various cancers, diabetes, and atherosclerosis [65]. There is some thought that oxidative stress may play a part in the progression of AD. The brain is more susceptible to oxidative stress than other organs due to its high energy consumption, and neurons are more likely to be affected by oxidative stress due to their higher metabolic rate [66]. It is thought that antioxidant factors act as a defense against ROS to prevent any injury. But when the ROS levels exceed that of the antioxidant activity, they attack cell macromolecules, destroy cell function, and induce neuronal apoptosis [67]. On the other hand, low levels of ROS stimulate neurons, leading neurons to be possibly protected from any damage [67]. NO radical is one example of a reactive nitrogen species (RNS) and is synthesized by nitric oxide synthase (NOS) [68].

NO is a type of gaseous signaling molecule that plays a critical role in many functions within the CNS, including neurotransmission, synaptic plasticity, memory function, and vasodilation of smooth muscle [69,70]. NO then stimulates cyclic guanosine monophosphate (cGMP), which begins a series of intracellular events, leading to vasodilation [70]. In the presence of oxygen and nicotinamide adenine dinucleotide phosphate (NADPH), the conversion of L-arginine to L-citrulline via NOS can take place, producing NO [69].

Three different isoforms of NOS are present within the body: neuronal nNOS, inducible iNOS, and endothelial eNOS. Each isoform possesses its own function, with the ability to work independently and together within various systems.

iNOS is a soluble enzyme that is able to exist in the cytosol [71]. It is different than the other two isoforms because it is not constantly present within cells and is only expressed when triggered or stimulated. Pro-inflammatory cytokines and/or bacterial lipopolysaccharides are common culprits [72]. iNOS activity is independent of Ca^2+^ and plays an important role in the regulation of neuroinflammation by allowing NO to function as a vasodilator as well as a stimulant in leukocyte adhesion to blood vessels in the endothelium [73].

eNOS is a non-soluble enzyme frequently located in the plasma or Golgi body membrane [71]. It is most commonly present within endothelial cells, though it has been found in various locations, such as platelets and epithelial kidney cells [74]. eNOS is also constitutively expressed, and its activity is regulated by Ca^2+^ [75]. Most NO is synthesized in the endothelial cells by eNOS, with its role in vasodilation well established.

Neuronal NOS (nNOS) is a soluble enzyme that can be found in the cytosol of cells [71]. It can be found both pre- and postsynaptically within the cytoplasm and membrane of each subpopulation in each major neuron type within their ventral cochlear nucleus [76]. nNOS is also found within smooth muscle and within the cells of the central and peripheral nervous systems [74]. It is constitutively expressed, and its activity and expression are regulated by Ca^2+^. nNOS is distributed across the brain and spinal cord, with the amount varying from region to region. The generation of NO in varying regions has a stimulatory effect on excitatory synapses, due to the relationship between ionotropic N-methyl-D-aspartate (NMDA) glutamate receptor, Ca^2+^, and nNOS. It has been proposed or hypothesized that NO produced by nNOS may only interact with localized surrounding subsynaptic divisions of the brain. Within the vast microvascular constructs of the brain, there are two mechanisms in place regulating cerebral blood flow (CBF): autoregulation and neurovascular coupling.

nNOS does not work alone, with a multitude of evidence suggesting eNOS also produces NO within the brain [77]. In vitro, the production of NO in the endothelium affects both synaptic plasticity, specifically in the hippocampus, which is a common occurrence seen in AD, as well as the membrane potential in the axons of the optic nerve [73,78]. nNOS seems to affect neurovascular coupling, which connects brief neural activity to modifications in CBF, whereas eNOS tends to affect autoregulation, which refers to the ability to sustain blood flow even with varying cerebral perfusion pressure. Extended exposure to increased eNOS activation leads to granulocyte adherence to blood vessels, dysregulation of vascular perfusion, and increased expression of pro-inflammatory responses, ultimately causing a thickening of the capillary basement membrane, neurodegenerative decline, and neuronal cell death, which are all seen within AD patients [73].Oxidative stress is defined as an imbalance between the production and accumulation of ROS in cells, as well as the capacity of the body to detoxify the products with antioxidants [65]. ROS are mainly produced in mitochondria during various processes like protein phosphorylation, apoptosis, and immunity, which are all dependent on ROS production [65]. Glial cells have been known to release ROS in response to immune stimuli [69]. Accumulation of ROS can lead to the onset or progression of various cancers, diabetes, and atherosclerosis [65]. It has been theorized that oxidative stress plays a part in the progression of AD, since the brain is more susceptible to damage caused by oxidative stress compared to other organs due to its high energy consumption. Neurons are more likely to be affected by oxidative stress due to their higher metabolic rate [66]. It is thought that antioxidant factors act as a defense against ROS to prevent any injury. But when the ROS levels exceed that of the antioxidant activity, they attack cell macromolecules, destroy cell function, and induce neuronal apoptosis [67]. On the other hand, low levels of ROS stimulate neurons, leading neurons to be possibly protected from any damage [67].

All three NOS isoforms appear in abnormal levels in AD patients, leading to elevated NO concentrations, possibly leading to an accumulation of ROS and worse oxidative stress. The high levels of NO are thought to be involved in the pathogenesis of AD by possibly directly or indirectly causing neuronal death [79]. Another pathway for NO synthesis is via nitrile, occurring primarily due to the presence of ischemic conditions. These conditions are a common trigger for AD development when the NOS output seems to have reached its maximum threshold [73,80]. Consequently, this allows for more NO to be produced even when the NOS pathways seem to be compromised [73].

## 4. Cardiovascular Risk Factors

There are many different risk factors that increase the likelihood of a person developing AD, with one of the more significant factors being cardiovascular risk factors, such as Type 2 diabetes. This association has led AD to be deemed in part a neuroendocrine disorder and given the moniker of “type 3 diabetes” [81]. Studies have shown a negative correlation between insulin expression and AD progression [82]. Patients with Type 2 diabetes have a higher risk of developing AD and vascular dementia, with correlations in changes in insulin and glucose in both disease states [83]. One study showed decreased levels of insulin-like growth factor binding protein-2 (IGBP-2) and increased levels of insulin-like growth factor 1 receptor (IGF-1R) in the temporal cortex of AD patients [83]. A longitudinal study of diabetic patients over a span of 4 years reported a decline in cognitive performance, suggesting a relationship between diabetes and cognition [84]. Diabetes mellitus type 2 is a risk factor of AD. Insulin resistance and a high level of glucose factor into increased morbidity [85]. Insulin resistance can lead to a disruption in glucose transport and then to weakened glucose metabolism; together, they are thought to cause neurodegeneration in AD [85].

Chronic high blood pressure, or hypertension, is another cardiovascular risk factor associated with cognitive decline. Hypertension is defined by a systolic blood pressure ≥ 140 mmHg or a diastolic blood pressure ≥ 90 mmHg [86]. One possible cause of hypertension is the failure of primary endothelial cilia to sense fluid shear stress, leading to NO deficiency [52]. Endothelial cilia in the vasculature are responsible for the production and release of NO [50]. So, if dysfunctional cilia are unable to detect blood flow mechanically, then NO will not be produced, leading to increased blood pressure.

A systematic review of four studies showed a direct association between hypertension and AD neuropathic change [87]. Hypertension in older adults leads to maladaptation of the cerebral circulation, causing a disruption in the blood–brain barrier (BBB) and oxidative stress [86]. This oxidative microvascular damage and BBB disruption caused by hypertension could worsen the progression of AD [86]. One study in Japan showed that increased blood pressure variability may be a significant risk factor for the development of AD [1]. Another study showed that elevated blood pressure in older adults is associated with plasma AD biomarker levels [88]. Like the Japan study, it was suggested that BP becomes variable over time due to arterial stiffness. Higher blood pressure causes stiffening and remodeling of large arteries because of the increased mechanical stress placed on the arterial walls [89]. This arterial stiffening can alter pulse wave dynamics, leading to a buildup and inconsistent flow of blood [88]. This may cause high flow pulsatility and damage cerebral microcirculation as the BBB is where most of the nutrient transfer, influx, and waste clearance occurs [88]. In a longitudinal study, midlife hypertension was associated with the amyloid beta plaque formations, development of neurofibrillary tangles, and low brain weight, which are all hallmarks of AD [90]. A multicenter study took this a step further and analyzed data from three observational european studies of cognitively unimpaired participants, looking at associations between blood pressure and AD biomarkers in cerebral spinal fluid (CSF) and Aβ positron emission tomography (PET) [91]. This study revealed that higher blood pressure measurements were associated with a larger amount of amyloid pathology [91]. It also showed a negative association between diastolic blood pressure and CSF levels of Aβ_42_ and p-tau181 but these associations went away after adjusting for CSF AB_40_ [91]. All these studies add support to high blood pressure being associated with an increased pathology of AD and amyloid deposition.

## 5. Small-Scale Vascular Manifestations

Vascular manifestations of AD occur on a small and large scale in the brain. Small-scale manifestations demonstrate how the brain is affected at the cellular level. Endothelial cells, which line the lumen of the cerebrovasculature, are important in controlling blood flow throughout the brain. They represent a component of the BBB that regulates the movement of molecules by transporters and tight junctions, as well as controls vasomotor function by dilation or constriction [92]. Endothelial dysfunction is associated with AD, even when adjusting for age and cardiovascular risks, which can also affect the endothelium [93]. The pathogenic relationship between cognitive decline in AD and vascular endothelial dysfunction is not clear but is thought to be due to Aβ [93,94]. Aβ has been shown to negatively impact each part of the NO/cGMP pathway. Both the fibrillar and oligomeric forms of Aβ suppressed the NO-mediated activation of soluble guanylyl cyclase, causing a decrease in cGMP production [69]. Also, after LTP induction in murine hippocampal slices, Aβ impeded the increase in cGMP immunoreactivity [69]. These pathways are not the only ones that are negatively impacted by Aβ, as it also disrupts Shh signaling and distorts primary cilia structure [49]. When mouse NIH3T3 fibroblast cells were treated with Aβ, there was a significant decrease in primary cilia length and in Shh downstream signaling [49].

Due to the accumulation of Aβ in the brain, there is an influx of ROS, which then generates oxidative stress and endothelial damage. The BBB, which is a barrier comprising endothelial cells, pericytes, tight junctions, and astrocytes, is important in regulating the movement of molecules to and from the brain. Due to the dysfunction of the endothelium and the tight junctions, the integrity of the BBB is disrupted, and the normal filtration of molecules is impacted, including the clearance of Aβ from the brain [93]. This also leads to an increase in inflammation and oxidative stress from the increased BBB permeability. These correlations between AD and dysfunction of the cerebrovasculature lead to the speculation that AD is a vascular disease [93].

A recent study found that patients with high amounts of amyloid burdens were found to have increased tau deposition, lower CBF, and more severe cognitive impairment [95]. CBF meets the metabolic demand of the brain by a narrow margin, with a small local energy reserve, making any reduction in CBF cause variability in brain function [95]. Proper CBF maintenance and regulation are important for brain function. In a healthy aging brain, the maximum CBF levels are reached around 4–6 years old, but decreases to 60–70% of that value by 50–60 years old [95]. As the body ages, cerebral blood vessels see an increase in collagen deposition and calcification in arterioles, causing increased thickness in vessel walls and a decrease in elasticity [95]. These changes can be correlated with increased levels of ROS, but these changes can be more severe in AD patients [95]. Both CBF and glucose metabolism are reduced, and vascular resistance is increased in patients with AD [96]. This reduction in CBF reaches over 50% in some brain areas, reducing the activity of the sodium/potassium pump [96]. The reduction in CBF is suggested to start early in preclinical AD, along with a fall in metabolism [96].

## 6. Large-Scale Vascular Manifestations

**Figure 3:**
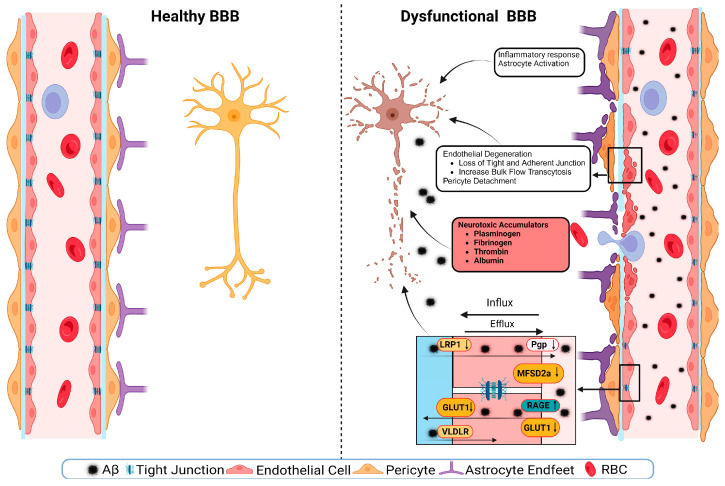
Healthy (left) vs. dysfunctional (right) blood brain barrier (BBB). BBB dysfunction may be caused by a disruption in the endothelial cells, leading to decreased tight junction expression and increased vascular permeability. Tight junction protein expression has also been reduced in the presence of Amyloid Beta 42 (Aβ_42_). Low-density lipoprotein receptor-related protein 2 (LRP2), Glucose Transporter Type 1 (GLUT1), very-low-density-lipoprotein receptor (VLDLR), Permeability Glycoprotein (Pgp), Major facilitator superfamily domain-containing protein 2 (MFSD2a), receptor for advanced glycation end products (RAGE), red blood cell (RBC). Figure is adopted with permission from [102].

Large-scale vascular manifestations demonstrate how the brain is affected by pathological changes. Cerebral amyloid angiopathy (CAA) involves the cerebrovascular deposition of Aβ in the cortical and leptomeningeal blood vessels [97]. This can cause increased vascular fragility and can affect perivascular drainage, which may contribute to the pathogenesis of AD [98]. One study showed that a decrease in the availability of eNOS leads to a higher cerebrovascular concentration of Aβ [99]. There is a high coincidence between AD and CAA. A strong correlation coincidence has been found between AD and CAA in a study of 404 brains [100]. However, AD and CAA do not necessarily manifest in the same way, as CAA is largely caused by blood vessel dysfunction in the form of hemorrhage due to loss of vessel integrity or ischemia caused by the loss of normal blood supply [98]. One common early trait of AD is leakage in the BBB. The BBB is formed by the endothelial cells that line the walls of the capillaries; this is the first principal barrier (Figure 3). The second layer is choroid plexus endothelial cells that face the cerebral spinal fluid, also known as the blood-cerebrospinal fluid barrier [101]. The third interface is the avascular arachnoid epithelium, which sits under the dura, encircling the CNS. The two ways a molecule can enter the capillaries and into the brain are through transport across the BBB or through tight junctions.

**Figure 4:**
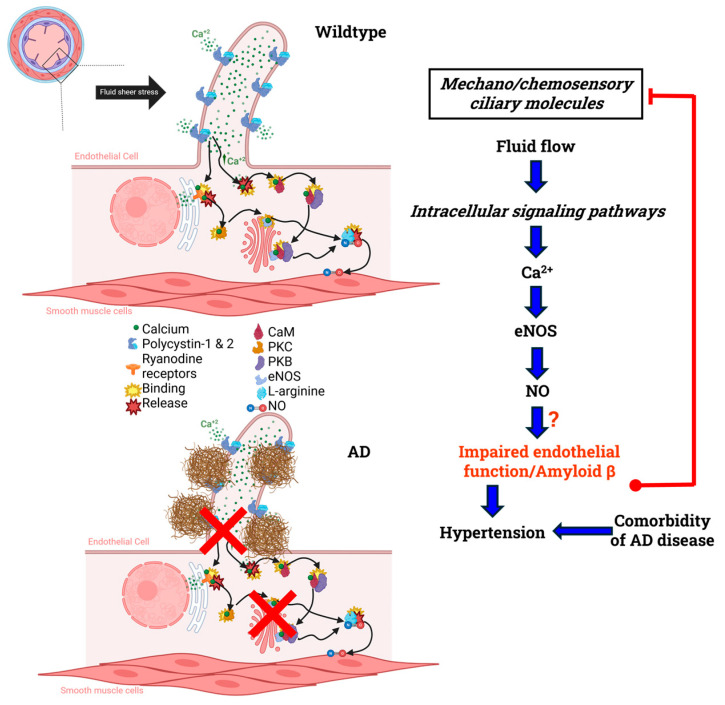
NO signaling in healthy vs. Alzheimer’s disease primary cilia. Primary cilia are sensory organelles that extend from the surface of endothelial cells into the blood vessel lumen, where they detect extracellular stimuli and convert them into intracellular biochemical signals, such as NO. In Alzheimer’s Disease Aβ accumulation can possibly disrupt downstream signaling pathways, leading to impaired vascular function and cognitive decline but the exact mechanism is still unclear. Created in part with BioRender Premium [111] (https://help.biorender.com/hc/en-gb/articles/17605447498269-BioRender-Premium accessed on 10 September 2025).

Whether it be through passive diffusion, solute carriers, ATP-binding cassette (ABC) transporters, or transcytosis, smaller, highly lipid-soluble, or polar molecules are able to pass through the BBB [101]. Tight junctions are the space between endothelial cells and lack intercellular clefts. Pericytes make up part of the basement membrane and regulate vascular permeability [103]. Astrocytes have protrusions that stick to the basement membrane of blood vessels, making it harder for harmful substances to enter [103].They express Aquaporin-4 (AQP-4), a water channel that facilitates water transfer [102]. These, along with low pinocytotic activity, make it very difficult for molecules to cross [104]. The BBB is an active barrier in a healthy brain. It inhibits the entry of compounds, maintains brain homeostasis, and provides the brain with oxygen and nutrients. In the BBB, the low-density lipoprotein receptor-related protein 1 (LRP1) plays a major role in maintaining Aβ homeostasis and mediates transport of Aβ across the BBB into circulation [105,106]. Surface LRP1 is expressed on the abluminal side of the endothelial membrane, which binds to cerebral Aβ and begins its clearance into the luminal side. Soluble LRP1 (sLRP1) circulates freely in plasma and sequesters free Aβ in circulation and in neurologically normal people. sLRP1 has been shown to sequester 70–90% of plasm Aβ [107]. In normally aging and AD models, LRP1 expression levels are reduced in the brain endothelial cells, which leads to higher Aβ levels in the brain. On the opposite side, the receptor for advanced glycation end products (RAGE) mediates the transport of Aβ from the bloodstream into the brain [108]. In AD, there is an increase in immunoreactivity of endothelial RAGE. Aβ induces various pathways that lead to the damage of cerebral endothelial cells. Aβ impairs the capability of cerebral endothelial cells to produce vasodilation, and this impairment is moderated by the production of ROS [109]. Aβ also triggers Ca^2+^ increases in endothelial cells by Transient Receptor Potential Cation Channel, Subfamily M, Member 2 (TRPM2) [110]. TRPM2 activation by Aβ is associated with substantial increases in intracellular Ca^2+^, leading to intracellular Ca^2+^ overload [110]. Figure 4 shows the possibility of Aβ binding to the luminal side of blood vessels and blocking primary ciliary function.

Barrier leakage indicates an increased amount of passage of bloodborne substances into the brain [104]. Oxidative stress and damaged perivascular Aβ clearance can cause degeneration of the neuronal vascular unit, leading to BBB leakage [104]. Emerging preclinical and early clinical evidence implicates eNOS deficits as a critical mechanistic link between neurovascular unit (NVU) dysfunction, BBB breakdown, and cognitive decline [112,113]. Mice with partial eNOS deficiency (*eNOS^+/−^*) develop early hypoperfusion, progressive BBB hyperpermeability, white-matter injury, microinfarcts, and age-dependent memory impairments and show greater BBB disruption under stressors such as hypoxia or chronic hypoperfusion, with worse cognitive outcomes when eNOS is reduced or absent [112,114]. On the other hand, restoration of endothelial NO signaling has been shown to mitigate these phenotypes across several experimental models [56,115,116], highlighting the therapeutic potential of restoring or enhancing endothelial NO signaling to stabilize the neurovascular unit, reduce amyloid burden, and protect cognitive function. Pericytes that are supposed to maintain the integrity of the BBB degenerate in AD [117]. When pericytes are injured, they release soluble platelet-derived growth factor receptor-β (PDGFRβ) [118]. This degeneration of pericytes is greater in AD patients with the apolipoprotein E4 (APOE4) gene, a major genetic risk factor of late-onset AD [117]. Astrocytes degenerate in AD, leading to a reduction in Aβ clearance, as the expression of AQP-4 is diminished [102].

For a few decades, scientists were not convinced that BBB leakage contributes to cognitive impairment. It was not until magnetic resonance imaging (MRI) techniques were combined with capillary markers to detect barrier leakage [104]. Dynamic contrast-enhanced MRI measures signal change after an intravenous injection of Gadolinium-based contrast agents (GBCA) [118]. An elevated transfer constant, K_trans_- the rate of GBCA extravasation from the bloodstream to the cerebral tissue- is an indicator of a dysfunctional BBB, as GBCAs do not cross the BBB. This imaging technique has sensitivity towards large and small changes in BBB functionality, so it has previously been used to look at patients with tumors or strokes [118]. One study used this technique to determine BBB leakage in patients with vascular mild cognitive impairment and found a higher leakage rate in various parts of the brain compared to age-matched controls [119]. Another study performed a cross-sectional study looking at multiple AD biomarkers in normal cognition (NC), mild cognitive impairment (MCI), and AD dementia patients [120]. The study showed higher K_trans_ rates in the AD patients than those with NC or MCI, suggesting BBB disruption in the AD patients, but showed no differences in Q-Alb [120]. The most commonly used clinical parameter to characterize barrier leakage is the CSF-serum ratio, also known as the Q value [104]. In healthy patients, protein levels of albumin are 100 to 200 times higher in the blood than in the CSF, as albumin originates in the blood [118]. But in patients with impaired BBB, their Q-Alb levels are elevated [104]. The same CSF-serum ratio can also be applied to immunoglobulin G, where a higher value can indicate increased BBB permeability [118]. Another CSF biomarker of BBB deterioration is PDGFRβ. CSF PDGFRβ is associated with neuroinflammatory markers and tau pathology and increases with age. One study looked at brain capillary damage using PDGFRβ as a CSF biomarker and dynamic contrast-enhanced MRI [121]. Their data suggest that brain capillary damage and BBB breakdown develop in the hippocampus of patients with early cognitive dysfunction, regardless of AD Aβ or tau biomarker changes [121]. This suggests that BBB breakdown is an early biomarker of cognitive dysfunction [121]. Another biomarker for BBB injury is Angiopoietin-2 (ANGPT-2) [118]. ANGPT-2 regulates endothelial permeability and angiogenesis. Endothelial cells release ANGPT-2 in response to harmful conditions like hypoxia, which results in decreased structural integrity, pericyte loss, and increased permeability of the BBB [118]. One analysis looked at ANGPT-2 in three cohorts of AD patients and healthy patients and found elevated levels of ANGPT-2 in patients with AD, as well as positive correlation of CSF ANGPT-2 to Q-Alb in cohort iii [122].

There are a few comorbidities of AD that relate to BBB leakage, as BBB leakage is a common pathological hallmark of both epilepsy and AD. Epilepsy is a brain disorder characterized by recurring seizures. Barrier leakage in epilepsy promotes seizures through a positive feedback loop that further progresses the condition [104]. These seizures propel barrier leakage that worsens neuroinflammation and future seizures.

In AD, the combination of BBB dysfunction, impaired cilia signaling, and reduced NO bioavailability acts synergistically to establish a vicious feed-forward loop that accelerates disease progression (Figure 4). BBB disruption allows peripheral inflammatory mediators and neurotoxic molecules to infiltrate the brain parenchyma, amplifying neuroinflammation [123,124]. This inflammatory milieu impairs the function of primary cilia on endothelial and neuronal cells, disrupting key signaling pathways such as Sonic hedgehog and Wnt that are essential for vascular stability and neuronal communication [125,126]. Ciliary dysfunction further exacerbates vascular dysregulation by impairing eNOS activity, leading to reduced NO production. Since NO is critical for vasodilation, cerebral blood flow regulation, and maintenance of BBB integrity, its deficiency worsens hypoperfusion and promotes additional barrier breakdown [127]. The resulting cycle barrier leakage driving inflammation, cilia impairment suppressing NO signaling, and NO deficiency fueling further vascular and neuronal injury, creates a self-reinforcing cascade that contributes to cognitive decline in AD.

## 7. Cilia as Potential Therapeutic Target

The primary cilium has gained attention as a prospective therapeutic target across a variety of human diseases due to its pivotal involvement in cellular signaling processes, mechanosensory properties, and its ability to modulate important signaling pathways. These diseases, caused by genetic and non-genetic mutations that disrupt primary cilia structure and/or function, include Bardet-Biedl Syndrome, Joubert syndrome, Polycystic kidney disease, some cancers, cardiovascular diseases, osteoarthritis, and neurodegenerative disorders such as AD [128]. Therapeutic approaches aim to restore or enhance ciliary function and/or structure, modulate the cilia-associated components, and target cilia-related downstream mechanisms to help end progression or cure the disease [129].

Primary cilia are involved in specific cellular signaling pathways relevant to AD. First, primary cilia are present on neurons and glial cells, assisting in maintaining neuronal health and proper brain function. Abnormalities within primary cilia can have detrimental effects on ciliogenesis and proliferation within the brain. For instance, Aβ accumulation can disrupt the Sonic Hedgehog signaling pathway and other pathways that are guided by primary cilia, potentially leading to AD [49,130]. Since Aβ has been shown to decrease cilia length, small molecules that restore cilia length may show promise as therapeutic solutions [47,48]. A few studies have identified various compounds and drugs that restore cilia length, including MI-181 in retinal pigment epithelial-1 cells, cevimeline in *Tg737^Orpk/Orpk^* cells, and Clofibrate, Sirolimus, and Dexamethasone in various cancer cell types [52,131,132].

As previously mentioned, AD is linked to oxidative stress, representing a dysregulation in the equilibrium between ROS production and the body’s capacity to detoxify or repair the molecular damage. Numerous reports underscore the role of ROS in regulating the structure of primary cilia in various disease or injury states, such as during kidney injury. Consequently, a growing body of evidence highlights a complex interplay between primary cilia and mitochondrial function [133]. Additionally, primary cilia within vascular endothelial cells contribute to blood pressure control by sensing fluid shear stress, triggering Ca^2+^ influx, and facilitating the release of NO, which induces vasodilation [71]. M3R is localized to the primary cilia of endothelial and cerebrovascular cells. In terms of eNOS activation, M3R activation increased cilia length and sensory function [52].

Emerging evidence suggests a collaborative role in the pathogenesis of AD, positing primary cilia as a potential therapeutic target for slowing down disease progression. Despite the evidence hinting at potential connections between primary cilia, oxidative stress, and NO bioavailability, a comprehensive understanding of the underlying mechanisms and their clinical implications requires further investigations.

## 8. Future Directions

With a significant number of unanswered questions about the pathogenesis of AD, there are a few different future directions for where to go from here. As of the writing of this report, there are only a few blood-based biomarkers specifically for AD in routine clinical use. Two that focus on endothelial dysfunction are ANGPT2 and vascular endothelial-Cadherin, both found in elevated levels in the CSF [118]. In AD, typically, there is a low amount of Aβ 1–42 in the CSF but elevated levels of tau and phospho-tau [134]. This pattern is being used in the diagnostic criteria of AD as an imaging biomarker in amyloid PET [134,135]. Multiple phosphorylated and non-phosphorylated tau forms are also being used as imaging biomarkers for Tau PET [135]. While these scans are very accurate, they are expensive and not widely accessible. Some scientists are turning to machine learning models to predict individual-level Aβ and *τ* PET positivity status [136]. By incorporating multimodal data from standard neurological work-up, they hope to identify individuals with biological AD, without needing to use PET scans [136]. A different model is being developed to look at Aβ and tau accumulation as part of a cellular phase of AD pathogenesis instead of a sequential pathway [137]. This model could change how scientists approach treatment and prevention [137]. Others believe that a time will come when polygenic risk scores can be combined with other risk factor health measures to create an individual risk score, similar to the Framingham cardiovascular risk factor [134]. This would allow high-risk individuals to be referred to better tests of AD pathology, with a range of treatments based on the patient’s state of disease [134]. In a similar fashion, it may be more effective to treat patients with a combination of therapies. While this method shows promise, experts in the field recognize that combination approaches present challenges in clinical trial design, regulatory requirements, and non-clinical model limitations [138]. Because this is a newer approach to treating AD, combination therapies have not made their way out of the trial stage. These combination therapies might address two core pathologies, two or more types of non-core tissue reactions, two or more types of non-AD pathologies, or a combination of all three [139]. One of the most studied combination drug therapies is the use of memantine and cholinesterase inhibitors, which has proven clinical efficacy in the treatment of moderate to severe AD [140]. Although no drugs on the market for AD currently target vascular changes, it is possible for combination therapies to target non-AD co-pathology. As hypertension is a cardiovascular risk factor of AD, cilia would make a novel therapeutic target against AD.

While there are a few FDA-approved drugs for the treatment of AD, scientists are working on better ways to deliver drugs to the BBB. Nanoparticles have been a breakthrough in passing drugs through the BBB for the potential to treat CNS diseases. The BBB prevents pathogens and toxins from entering the brain and only allows small molecules to be passively diffused. This leads to limits in the delivery of effective drugs and reduces therapeutic efficiency [141]. A few preclinical studies have employed the use of nanoparticles to deliver FDA-approved drugs for the treatment of neurological diseases. Scientists in India prepared self-assembling nano scaffolds loaded with memantine that were subjected to in vitro release and found to have 91% drug diffusion after 3 days [142]. Memory-impaired mice were also given various dosages of the memantine-loaded PLGA self-assembled scaffolds intrathecally and found a significant reduction in pro-inflammatory cytokines [142]. A different approach was taken with a nanoemulsion loaded with memantine for an intranasal delivery to circumvent the BBB [143]. Biodistribution results showed a higher uptake of formulation in rats administered intranasally compared to intravenously [143]. Although none exist right now, it would be an interesting direction to use combination therapies or nanoparticles to target and restore cilia dysfunction in relation to AD. While all these prospects sound ambitious, only time will tell.

## 9. Conclusions

Overall, it has been shown that there is a lot of overlap in the symptoms of AD and cardiovascular disease in how NO and primary cilia play a role. Primary cilia are sensory organelles that detect changes in blood flow. The cilia then respond to changes in blood pressure by activating a signaling pathway that regulates and synthesizes the release of NO. NO stimulates the production of cGMP, which initiates a cascade of intracellular signaling events that lead to the relaxation of smooth muscle cells and result in vasodilation. When dysfunctional cilia fail to detect fluid shear stress, endothelial production of nitric oxide (NO) decreases, potentially contributing to elevated blood pressure. Hypertension is a risk factor for the development of AD [52]. Further investigations need to be performed to fully understand the possible connection between primary cilia, NO, and AD.

## Figures and Tables

**Table 1:** Various cell types with motile or primary cilia and their specialized function.

	Cell Type	Function
Motile	Ependymal cells	Propel cerebrospinal fluid [23]
	Choroid plexus epithelium	Regulate cerebral spinal fluid production [24]
	Tracheal epithelial cells	Remove foreign substances from the body [25]
	Oviductal epithelium	Facilitate transport of oocyte, gamete, and embryo [26]
	Nasal epithelial cell	Mucociliary clearance [27]
Nonmotile	Neural stem cells	Regulation of Neural stem cells, especially in the ventral region of the ventricular–subventricular zone [28]
	Neurons	Regulate cognitive function, metabolism, and mood state [29]
	Astrocytes	Regulates astrocyte morphology and intracellular signaling balance [30]
	Osteoblasts	Participate in osteoblast alignment, differentiation, and polarization, as well as bone formation [31]
	Osteocytes	Act as mechanical sensors and bend in response to pulses of extracellular fluid that are generated during running and walking [32]
	Chondrocytes	Mechanotransduction [31]
	Dental pulp stem cells	IFT80 ciliary protein helps regulate Dental pulp stem cells differentiation [33]
	Vascular endothelial cells	Calcium-dependent mechanosensors that sense blood flow [34]
	Renal tubule epithelial cells	Mediate the mechanosensation of extracellular urine flow [35]
	Endocardial cells	Mediate upregulation of *the KLF2* gene expression and succeeding activation of the Notch signal pathway [36]
	Vascular smooth muscle cells	Mediate extracellular matrix-protein sensing and fluid-flow-induced mechanosensing [37]
	Olfactory epithelial cells	Odorants bind to olfactory receptors to start the olfaction cascade [25]
	Stereocilia	Mechanoelectrical transduction- converting physical force to an electrical signal [38]
	Kinocilia	Mediate hair cell morphogenesis and planar cell polarity [39]
	Cholangiocytes	Mechanosensory, chemosensory, and osmosensory functions to regulate cholangiocyte proliferation [40]
	Retinal photoreceptor cells	Connects inner and outer segments- the cellular nucleus to the photopigment [41]
	Retinal ganglion cells	Facilitate regenerative responses to Insulin-like Growth Factor-1 [42]

## Data Availability

No new data were created or analyzed in this study. Data sharing is not applicable to this article.

## Abbreviations

The following abbreviations are used in this manuscript:

AD	Alzheimer’s Disease
NO	Nitric oxide
Aβ	Amyloid beta
APP	Amyloid precursor protein
MPRAGE	Magnetization Prepared Rapid Gradient Echo
NLRP3	nucleotide-binding domain, leucine-rich–containing family, pyrin domain–containing-3
BACE-1	Beta-Site Amyloid Precursor Protein Cleaving Enzyme 1
Ca^2+^	Calcium
Shh	Sonic Hedgehog
CaM	Calmodulin
PKC	Protein kinase C
PKB	Protein kinase B
eNOS	Endothelial nitric oxide synthase
Hh	Hedgehog pathway
GPCRs	G-protein-coupled receptors
SSTR3	Somatostatin Receptor 3
5-HT6	5-hydroxytryptamine
MR	Muscarinic acetylcholine receptor
AChEI	Acetylcholinesterase inhibitors
ACh	Acetylcholine
M1R	Muscarinic receptor 1
M3R	Muscarinic receptor 3
p-eNOS	Phosphorylated endothelial nitric oxide synthase
CNS	Central nervous system
ROS	Reactive oxygen species
RNS	Reactive nitrogen species
NOS	Nitric oxide synthase
cGMP	Cyclic guanosine monophosphate
NADPH	Nicotinamide adenine dinucleotide phosphate
nNOS	Neuronal nitric oxide synthase
iNOS	Inducible nitric oxide synthase
NMDA	N-methyl-D-aspartate
CBF	Cerebral blood flow
IGBP-2	Insulin-like growth factor binding protein-2
IGF-1R	Insulin-like growth 1 factor
BBB	Blood–brain barrier
CSF	Cerebrospinal fluid
PET	Positron emission tomography
CAA	Cerebral amyloid angiopathy
LRP2	Low-density lipoprotein receptor-related protein 2
GLUT1	Glucose Transporter Type 1
VLDLR	very-low-density-lipoprotein receptor
Pgp	Permeability Glycoprotein
MFSD2a	Major facilitator superfamily domain-containing protein 2
RAGE	Receptor for advanced glycation end products
RBC	Red blood cell
ABC	ATP-binding cassette
AQP-4	Aquaporin-4
LRP1	Low-density lipoprotein receptor-related protein 1
sLRP1	Soluble low-density lipoprotein receptor-related protein 1
TRPM2	Transient Receptor Potential Cation Channel, Subfamily M, Member 2
NVU	Neurovascular unit
APOE4	Apolipoprotein E4
MRI	Magnetic resonance imaging
GBCA	Gadolinium-based contrast agents
NC	Normal cognition
MCI	Mild cognitive impairment
ANGPT-2	Angiopoietin-2
